# Rapid Fabrication of Cell-Laden Microfibers for Construction of Aligned Biomimetic Tissue

**DOI:** 10.3389/fbioe.2020.610249

**Published:** 2021-01-18

**Authors:** Bingchuan Lu, Mingfeng Li, Yongcong Fang, Zibo Liu, Ting Zhang, Zhuo Xiong

**Affiliations:** ^1^Biomanufacturing Center, Department of Mechanical Engineering, Tsinghua University, Beijing, China; ^2^Biomanufacturing and Rapid Forming Technology Key Laboratory of Beijing, Beijing, China; ^3^“Biomanufacturing and Engineering Living Systems” Innovation International Talents Base (111 Base), Beijing, China

**Keywords:** wet spinning, cell-laden microfiber, core-sheath microfiber, hollow microfiber, microfiber assembling

## Abstract

Bottom-up engineering of tissue constructs is being rapidly developed and broadly applied in biomanufacturing. As one type of building block, cell-laden microfibers are promising for reconstruction of oriented structures and functions of linear tissues, such as skeletal muscles, myocardia, and spinal cord tissues. Herein, we propose wet-spinning method with agitating collection, wherein alginate-based material is extruded into an agitated CaCl_2_ bath with a magnetic rotor acting as the microfiber collector. By applying this method, we achieve rapid fabrication and oriented collection of hydrogel microfibers with diameters ranging from 100 to 400 μm. In addition, we encapsulate myoblasts in the hydrogel to form cell-laden microfibers, which show a high viability (more than 94%) during *in vitro* culture. Moreover, the method allows to fabricate of cell-laden core–sheath microfibers and hollow microfibers. We also fabricate 3D constructs using various methods of microfiber assembly like weaving and braiding. The assembling results suggest that the proposed method is a promising technology for bottom-up engineering of aligned biomimetic tissue constructs.

## Introduction

The modular approach of bottom-up engineering for tissue constructs has been widely developed in recent years (Nichol and Khademhosseini, 2009; Nie and Takeuchi, 2018). To resemble the functional or structural tissue units of the native tissues or organs, such as muscle fibers, blood vessels, and renal corpuscles, microtissues have been designed, produced, and assembled into the target 3D constructs (Elbert, 2011). The bottom-up approach allows to generate bioengineered functional tissues with tunable microarchitectures, cell distributions, and precisely engineered properties (Gauvin and Khademhosseini, 2011).

**Figure 1:**
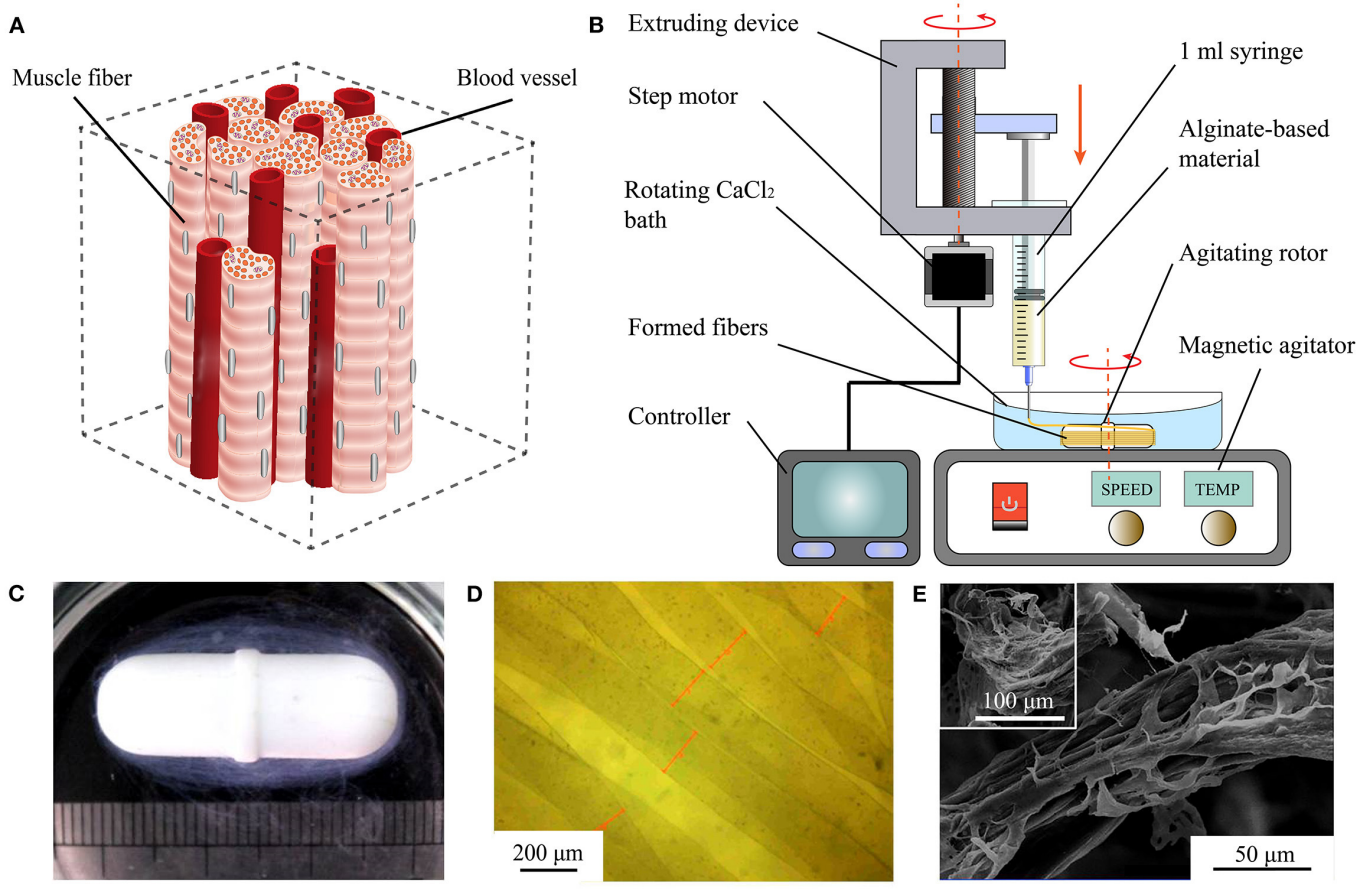
Rapid fabrication and observation of microfibers. **(A)** Simplified muscle tissue consisting of muscle fibers and blood vessels. **(B)** Diagram of proposed WSAC to produce alginate-based microfibers. **(C)** Optical image of hydrogel microfibers winding around rotor after forming. **(D)** Microscopic image of hydrogel microfibers with regular shapes. **(E)** Images from scanning electron microscope of freeze-dried alginate-fibrin microfibers.

Various building blocks such as cell sheets (Matsuda et al., 2007), cell spheroids (Lee et al., 2016), and cell-laden hydrogel blocks (Jiang et al., 2016) have been prepared and assembled into 3D structures using bottom-up engineering. Among them, cell-laden microfibers (CLMs) can directly reconstruct oriented hierarchical structures, such as muscle fibers, nerve networks, ligaments, and tendons (Onoe and Takeuchi, 2015). Consider a skeletal muscle that consists of individual muscle fibers arranged in parallel and contains branches of blood vessels (Levenberg et al., 2005). Obtaining aligned muscle fibers that can be assembled into organized muscle bundles is essential for engineering functional skeletal muscle tissue (Gholobova et al., 2020). Even if the native muscle tissue unit is simplified as an aggregation comprising aligned muscle fibers and blood vessels (Figure 1A), it remains difficult to stably and efficiently engineer that type of tissue construct, which has inner oriented structures and microchannels, by techniques such as 3D bioprinting (Kang et al., 2016) and micropatterning (Chen et al., 2015).

Unlike other techniques, the linear structure obtained from CLMs may enable the reconstruction of aligned tissue. Moreover, CLMs facilitate cellular activities like spreading (Yamada et al., 2012), orientation (Yang et al., 2017a), and functionalization (Daud et al., 2012), especially for tissues relying on aligned structures for their function. CLMs obtained from cell electrospinning promote the elongation and alignment of myoblasts, and the fibrous structure on the electrospun scaffolds induce cell–cell interactions in a unidirectional array as well as myotube formation and fusion (Yeo and Kim, 2018). Neuron cells seeded on flat alginate fibers have been interconnected by neurite bundles along the length of the fibers, whose aligned neuron cell cultures suggest their applicability for nerve regeneration (Kang et al., 2012). After 3 days of culture, meter-long microfibers encapsulated with primary rat cardiomyocytes have shown spontaneous 0.5–1 Hz contractions that can actuate the entire fiber (Onoe et al., 2013).

Heterogeneous structures and perfusion channels have been important for *in vitro* fabrication of biomimetic tissue (Ashammakhi et al., 2019). In fact, the formation of channels that transport oxygen and nutrients for cells in large-scale hydrogel structures has allowed to overcome limited diffusion distances of 150–200 μm (Lovett et al., 2009; Sharma et al., 2019). Fibers with multiple materials or hollow structures have also been studied. Kang et al. (2011) developed a microfluidic system that can continuously create microfibers with tunable morphological, structural, and chemical features. Zuo et al. (2016) fabricated composite hollow microfibers containing a double cell layer using a microfluidic system, thus establishing a promising alternative for complex tissue regeneration. Ouyang et al. (2017) proposed a generalizable strategy to fabricate various types of microfibers including core–sheath, heterogeneous, and hollow structures.

Regarding the fabrication of the abovementioned types of fibers, various biomaterials have been used, including alginate (Shin et al., 2007), chitosan (Lee et al., 2010), and GelMA (Zuo et al., 2016), resulting in different biocompatibility and mechanical properties. Among them, alginate is the most widely used biomaterial given its excellent formability and mechanical properties. In contrast, unlike melt spinning (Zuo et al., 2013) or electrospinning (Bhardwaj and Kundu, 2010), fabrication techniques such as microfluidic spinning and wet spinning are cell-friendly and stable during fabrication of hydrogel microfibers (Yang et al., 2017b), and the diameter of the wet-spinning microfibers can be easily adjusted by controlling the process parameters. However, most processes to fabricate single-layer, double-layer, and hollow CLMs disorderly form the microfibers in the liquid, thus hindering the subsequent assembly (Zuo et al., 2016; Wang et al., 2019). Various wet-spinning processes have been devised to collect pure microfibers without cells (Lavin et al., 2012; Yang et al., 2017b). Nevertheless, an integrated method to realize rapid fabrication, oriented collection, and assembling of various CLMs remains unavailable.

We propose the rapid fabrication of various CLMs for constructing aligned biomimetic tissue. In the proposed wet spinning with agitating collection (WSAC) method targeting the fabrication of CLMs, alginate-based material is continuously extruded into an agitating calcium chloride (CaCl_2_) solution and spun into microfibers collected with a magnetic rotor acting as microfiber collector. We evaluate various factors influencing the fabrication, such as crosslinking between alginate and calcium ions, swell effect, and flow effect. In addition, we encapsulate mouse myoblasts (C2C12) into the fabricated CLMs to study the cell behavior. Furthermore, we fabricated heterogeneous hollow CLMs with and without the C2C12 cells for their analysis. Finally, we report the assembly of CLMs into constructs, which are evaluated and will be used in future studies related to bottom-up tissue engineering.

## Materials and Methods

### Material Preparation

To fabricate the CLMs, we used sodium alginate from brown algae (Sigma-Aldrich, USA), calcium chloride anhydrous (Beijing Chemical Works, China), gelatin from porcine skin (Sigma-Aldrich, USA), bovine fibrinogen and thrombin (Shanghai Yuanye Bio-Technology, China), phosphate-buffered saline, fetal bovine serum (Biological Industries, Israel), high-glucose Dulbecco's modified eagle medium (H-DMEM), 0.25% Trypsin-EDTA, GlutaMAX™, MEM non-essential amino acids, and penicillin–streptomycin (Gibco, USA).

We used C2C12 myoblast cells purchased from the China Infrastructure of Cell Line Resources in Beijing. The cells are cultured in H-DMEM with 10%(v/v) fetal bovine serum, 1%(v/v) GlutaMAX™, 1%(v/v) MEM non-essential amino acids, and 1%(v/v) penicillin–streptomycin. Then, the cells are maintained in standard conditions (37°C, 5% CO_2_) and passaged until 80% confluence.

### Proposed WSAC

The developed WSAC system consists of an extruding part and a receiving part (Figure 1B). The extruding part is composed of a 1 ml syringe, a syringe pump, and a controller. Alginate-based hydrogel solution is loaded into the syringe and extruded into a calcium chloride (CaCl_2_) solution from the needle under the pressure applied by the syringe pump. The extrusion speed and volume of the hydrogel are adjustable. The receiving part is composed of a CaCl_2_ bath, an agitating rotor, and a magnetic agitator. Under the stirring action of the rotor, a steady flow field is formed in the CaCl_2_ bath. Alginate extruding from the needle is crosslinked with divalent calcium ions and forms microfibers winding around the rotor. After fibrinogen is added to the alginate solution to improve cell affinity, the formed microfibers are immersed in a thrombin medium to convert fibrinogen into fibrin.

### Fabrication of Hydrogel Microfibers

To improve cell viability and forming properties, the concentration of alginate used in bioprinting is generally <5% (w/v) (Gungor-Ozkerim et al., 2018), and it was selected as 1.25, 2.5, and 3.75%(w/v) in our study. We generate a 5%(w/v) sodium alginate solution by dissolving sodium alginate powder in sterile deionized water. By changing the mixing ratio of the sodium alginate solution and H-DMEM, we obtain materials with sodium alginate concentrations of 1.25, 2.5, and 3.75%(w/v). In addition, 2.5 and 5%(w/v) CaCl_2_ solutions are prepared with deionized water and 0.22 μm filtered before use. We also use needles with different sizes: 25G (inner diameter: 260 μm), 27G (inner diameter: 210 μm), 30G (inner diameter: 160 μm), and 32G (inner diameter: 110 μm). The extruding speeds of the pump for the experiments are 4, 6, 8, and 10 mm/s. We adjust the magnetic agitator to different agitating intensities of 20, 40, and 60%.

From the proposed WSAC process, we systematically evaluate the fabrication of hydrogel microfibers considering the microfiber diameter and mechanical properties.

#### Microfiber Diameter Analysis

We capture images of the microfibers using a VHX-500 digital microscope (KEYENCE, Japan) to characterize their morphology and measure their diameter. At least 10 samples of microfibers fabricated under different conditions are captured.

In the first place, we obtain the diameter of the microfibers fabricated with different material concentrations and needle sizes to plot bar charts for analyzing the relation between the material concentration and diameter. Secondly, the extrusion of hydrogel was mainly affected by the flow rate *Q* of the material and cross-sectional area *A* of the needle. The flow rate was determined by moving speed *v*_0_ of the extrusion module, whereas the cross-sectional area was determined by inner diameter *d*_0_ of the needle. Therefore, we calculated the velocity of flow *V* using Equation (1), for *d*_0_ and *v*_0_ expressed in micrometers (μm) and meters per second (m/s), respectively. Moreover, we calculated the shear rate $\dot{\gamma }$ of the hydrogel passing through the needle by using Equation (2), where *n* is the power index of the sodium alginate solution. Alginate hydrogels are shear-thinning fluids with value of *n* between 0.4 and 0.5 (Rezende et al., 2009) (we set *n* to 0.45 in this study).

(1) $$\begin{array}{r} V=\frac{Q}{A}=2.2\frac{v_{0}}{d_{0}^{2}}\times 10^{4}, \end{array}$$

(2) $$\begin{array}{r} \dot{\;\gamma }=\left(\frac{3n+1}{4n}\right)\frac{8V}{d_{0}}=23.0\frac{v_{0}}{d_{0}^{3}}\times 10^{10} \end{array}$$

Then, we obtain the diameter of the microfibers fabricated with different extruding speeds and needle sizes to analyze the relation between the shear rate and diameter. Moreover, we generated the rotating flow field by the stirring of the magnetic rotor, which determined the formation and movement of the CLMs. Thus, we obtain the diameter of microfibers fabricated at different agitating intensities of the agitator to analyze the relation between the rotating strength and diameter. Due to the limited diffusion distance, microfibers with diameter of around 200 μm are more suitable for cell growth. Thus, we fabricate microfibers with two different parameters, 27G at 8 mm/s and 30G at 6 mm/s, to determine the forming stability of the process.

#### Mechanical Analysis

**Figure 2:**
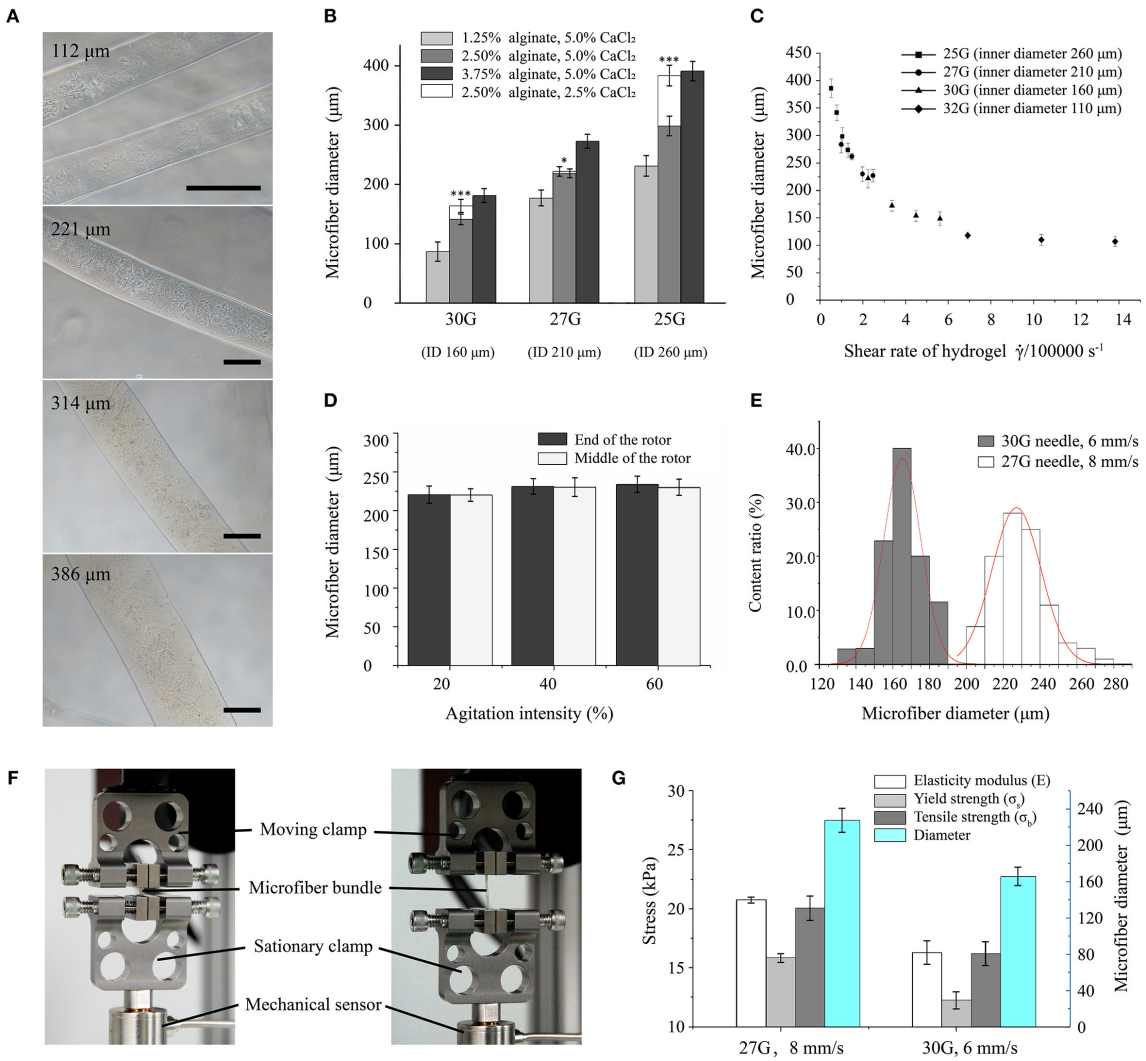
Morphological and mechanical characterization of hydrogel microfibers. **(A)** Microscopic images of microfibers with diameters from 100 to 400 μm (scale bar: 100 μm). **(B)** Microfiber diameter according to forming conditions (* and *** indicate significant difference between two types of fibers crosslinked by 5.0 and 2.5% CaCl_2_, respectively). **(C)** Microfiber diameter according to shear rate. **(D)** Microfiber diameter according to agitation intensity. **(E)** Distributions of microfiber diameter under different forming conditions. **(F)** Stretching setup for microfiber bundles. **(G)** Mechanical properties of microfiber bundles.

The mechanical properties of microfiber bundles fabricated under the two abovementioned conditions (27G at 8 mm/s and 30G at 6 mm/s) are tested. First, we cut the obtained loop microfiber bundles into 12–16 mm long bundles with an approximate diameter of 2.5 mm. A mechanical stretcher (ElectroForce 3200, BOSE, USA) and load cell (WMC-10C-456, BOSE, USA) are used to test the tensile properties of the samples. The microfiber bundles are clamped to a fixture and the effective tensile length is about 4 mm. Then, the bundles are stretched at 12 mm/min to a strain of 300% (i.e., 12 mm), in which local fracture occurred (see Figure 2F). The tension and displacement data collected by the sensor during tension are used to calculate the mechanical properties of the microfiber bundles. Elastic modulus was calculated from the linear slope of the stress-strain curve.

### CLM Fabrication

To fabricate the cell-laden hydrogel microfibers using the proposed approach, 3%(w/v) alginate solution and 3%(w/v) fibrinogen solution (both prepared in H-DMEM) are warmed at 37°C for 20 min and mixed in a ratio of 5:1. In addition, the 5%(w/v) CaCl_2_ solution is prepared with deionized water and 0.22 μm filtered before use.

Then, C2C12 cells are detached by 0.25% trypsin-EDTA solution, centrifuged turning to sedimentation, and evenly resuspended in the hydrogel solution. There are approximately 5.0 × 10^6^ cells in 1 ml of material. The cell-laden hydrogel microfibers are then fabricated using the mixture of C2C12 cells and moved into a six-well plate. Subsequently, the CLMs are immersed in 40 U/ml thrombin medium for 15 min at 37°C for the fibrinogen to be converted into fibrin. After the fibrinogen is crosslinked, the thrombin solution is removed, and the culture medium is added. The samples are cultured continuously, and the medium is refreshed every 2 days.

We observe the cell viability in the fabricated CLMs using fluorescent live/dead staining. Specifically, 2 μM calcein-AM (Dojindo, Japan) and 4 μM propidium iodide (Dojindo, Japan) are used to stain live cells (green) and dead cells (red) for 5 min at room temperature, and a laser scanning confocal microscope is used for observation (Z2; Nikon, Japan). We conduct staining tests on days 0, 1, and 3 of culture. The cell viability is determined using the count/size tool of Image-Pro Plus (Media Cybernetics, USA) by dividing the total number of cells by the number of green-stained cells. More than 10 random fields are counted per sample.

### Core–Sheath and Hollow CLM Fabrication

We use a coaxial WSAC system combining two extruding parts, a coaxial needle, and a receiving part. Hydrogel materials are extruded into the coaxial needle with a core (27G) and a sheath (20G) under the extrusion of two independent syringe pumps. Then, the CLMs are formed and collected in the rotating bath. Core–sheath microfibers are fabricated with 2.5% alginate for the two layers, and the core and sheath are added with different fluorescent particles (airbrush colors, Createx, USA), namely, green and red, respectively. Hollow microfibers are fabricated with 2.5% alginate (with added red particles) in the sheath and 5% gelatin (with sacrificial ink) in the core at 20°C. The temperature is then increased to 37°C to remove the gelatin after formation. The fabrication is also performed with labeled cells.

Green fluorescent dye (CellTracker™ Green CMFDA, Thermo Fisher Scientific, USA) and red fluorescent dye (CellTracker™ Red CMPTX, Thermo Fisher Scientific, USA) are used to stain the live cells independently and track their position. The culture medium is removed from the cells, and 5 μM of the staining solution is added into the culture flask and incubated at 37°C for 30 min. After fabrication, the samples are observed with the laser scanning confocal microscope to track the cell location.

### Assembly of 3D Constructs Using CLMs

We also design and fabricate bunch constructs based on microfiber assembly. The bunch loop is obtained from the rotor after the formation. We obtain microfiber bunches by cut the loop open. Then, the microfiber bunches are served as modules and manually assembled at different angles, like crisscross or helix, to form different constructs.

### Statistical Analysis

We analyzed the experimental data using OriginPro 2017 (OriginLab, USA) with results expressed as mean ± error. Each experiment was repeated at least three times. The Student's *t*-test was conducted to compare between two experimental groups with statistical significance corresponding to *p* < 0.05.

## Results

### Morphology of Hydrogel Microfibers

We fabricated alginate-based hydrogel microfibers using the proposed WSAC system (Figure 1B). Given the rapid crosslinking reaction between the sodium alginate and calcium ions, the alginate-based hydrogel began to crosslink once it was extruded into the solution through the needle. Under the action of the flow field, the microfibers were continuously generated and collected as expected. After the extrusion was completed, the microfibers wound around the rotor, as shown in Figure 1C. When disassembled in a phosphate-buffered saline solution, we obtained the bundle of hydrogel microfibers arranged along the same direction. When removed from the phosphate-buffered saline solution, the wetted microfibers were observed and measured under the digital microscope (Figure 1D). The images indicated relatively regular shapes and smooth surfaces of the fabricated microfibers. Upon freeze-drying, the microfibers appeared to be wizened visually and displayed an oriented topography, which was possibly caused by the extruding process. Furthermore, scanning electron microscope showed that phase separation occurred in the microfibers containing fibrin, leading to a special dissevered microstructure, in which dried solid alginate was inlaid and coated with floccus fibrin (Figure 1E).

### Characterization of CLM Fabrication

#### Influence of Process Parameters on CLM Diameter

##### Concentration of Materials

The diameter of the formed microfibers varied with the concentration of material. Microfibers with diameters ranging from 100 to 400 μm showed a well-distributed shape and smooth surface, as shown in Figure 2A. The relationship between material concentration and microfiber diameter is shown in Figure 2B. In general, the diameter mainly depends on the size of the needle, which increases with the increasing needle inner diameter. In addition, the microfiber diameter increases with increasing concentration of sodium alginate. The concentration reduction of CaCl_2_ solution also increases the microfiber diameter, especially for high concentration of sodium alginate. Moreover, the effect of the concentration of crosslinking medium is especially clear when using a large needle. Typically, the microfibers achieved a thinner diameter than the inner diameter of the needle with a low concentration of alginate, possibly due to the flow dragging.

##### Shear Rate

The relation between the microfiber diameter and shear rate was shown in Figure 2C. With a range from 100 to 400 μm, the microfiber diameters were similar to the inner diameters of the different needles. As the shear rate increased, the diameter decreased exponentially until converging and stabilizing.

##### Agitating Intensity

Figure 2D shows the relation between the agitating intensity of the magnetic stirrer and microfiber diameters. For agitating strength increasing from 20 to 60%, the diameter of the microfibers remained stable. Besides, the microfiber diameters at either the end or the middle of the rotor were almost equal.

##### Formation Stability

The diameters of microfiber fabricated with the first set of parameters (i.e., 27G at 8 mm/s) were distributed following a mostly normal distribution from 200 to 250 μm with a mean of 227 μm. For another set of parameters (i.e., 30G at 6 mm/s), thinner microfibers with a diameter of 145 to 185 μm were obtained, with the mean diameter decreasing to 165 μm. Figure 2E shows that the microfiber diameters obtained from the 30G needle had a smaller dispersion, thus being more uniform than those obtained from the 27G needle.

#### Mechanical Properties of Microfiber Bundle

The mechanical properties of the fabricated microfiber bundles were shown in Figure 2G. The microfiber bundle fabricated with the first set of parameters (i.e., 27G at 8 mm/s) exhibited better properties than that fabricated with the second set (i.e., 30G at 6 mm/s). The elastic modulus, yield strength, and tensile strength of the first bundle were 20.76 ± 0.26, 15.86 ± 0.37, and 20.06 ± 1.03 kPa, respectively, whereas those of the second bundle were 16.31 ± 0.98, 12.27 ± 0.73, and 16.21 ± 1.01 kPa, respectively. Hence, the property values of the first bundle were 20–30% higher than those of the second bundle. These results suggest that microfiber bundles consisting of microfibers with large diameters have higher elastic modulus and can withstand stronger stretching than their thinner counterparts.

### CLM Characterization

**Figure 3:**
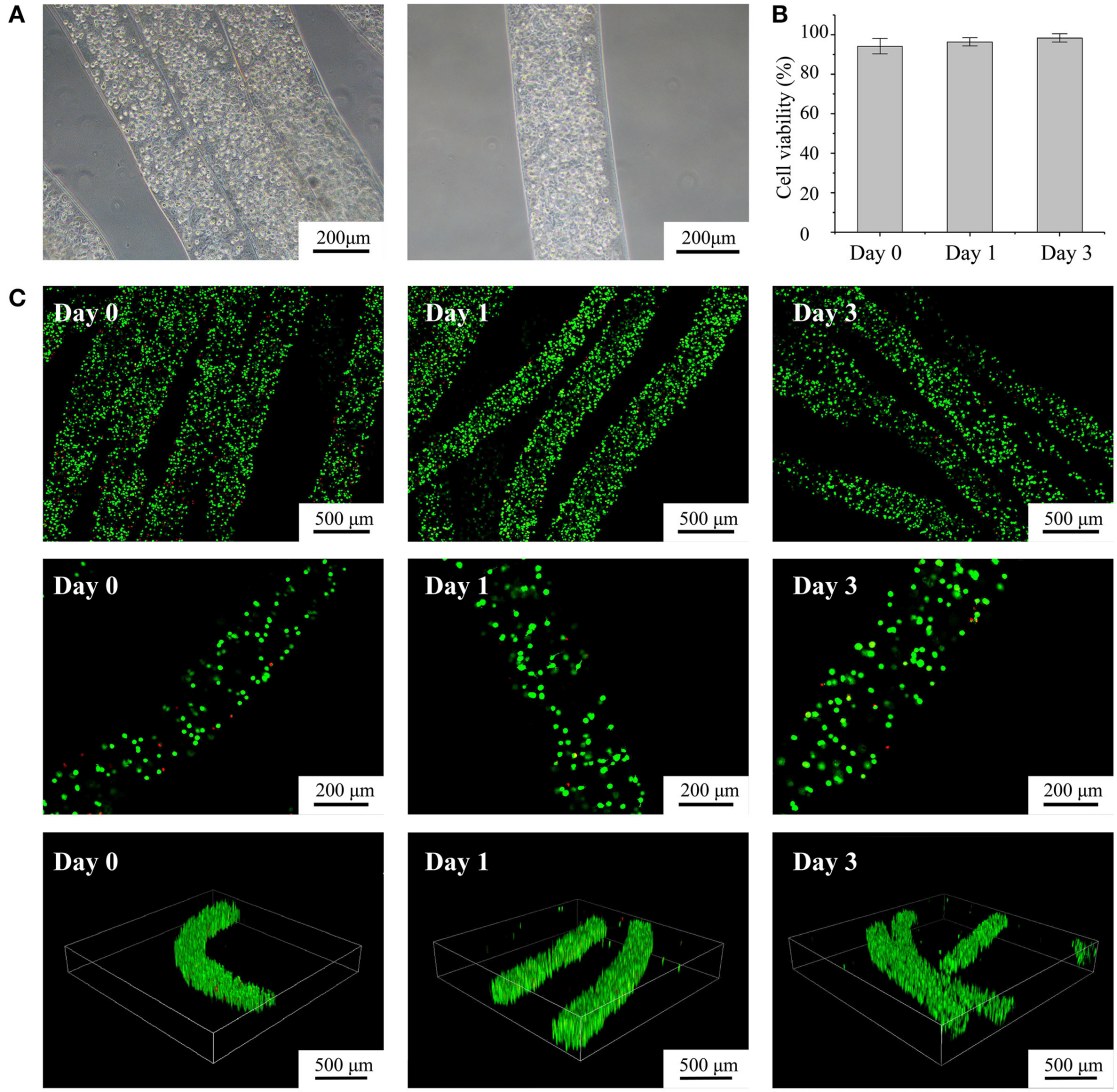
Characterization of fabricated cell-laden microfibers (CLMs). **(A)** Microscopic images of CLMs in bunch construct and individually. **(B)** Cell viability according to time of culture. **(C)** Confocal images of cell-laden C2C12 microfibers stained with calcein-AM/propidium iodide after 0, 1, and 3 days of culture at varying magnifications and 3D views.

We fabricated alginate-fibrin microfibers containing a high density of C2C12 cells, as shown in Figure 3A. The cells were uniformly distributed in the microfibers, which had an approximate diameter of 200 μm, being suitable for transporting oxygen and nutrients. Figure 3C shows the fluorescent live/dead staining results, where viable cells were stained in green by calcein-AM, and dead cells were stained in red by propidium iodide. By counting the number of viable and dead cells at 10 random regions, we obtained cell viability values at days 0, 1, and 3 of 94.2, 96.4, and 98.2% (Figure 3B), respectively. Thus, the C2C12 cells were highly viable during *in vitro* culture. The 3D structure of the microfibers during the culture period also demonstrated the high cell density and viability. Moreover, the alginate-fibrin microfibers maintained a stable shape during culture, and their diameter did not increase considerably.

### Characterization of Cell-Laden Core–Sheath Microfibers and Hollow Microfibers

**Figure 4:**
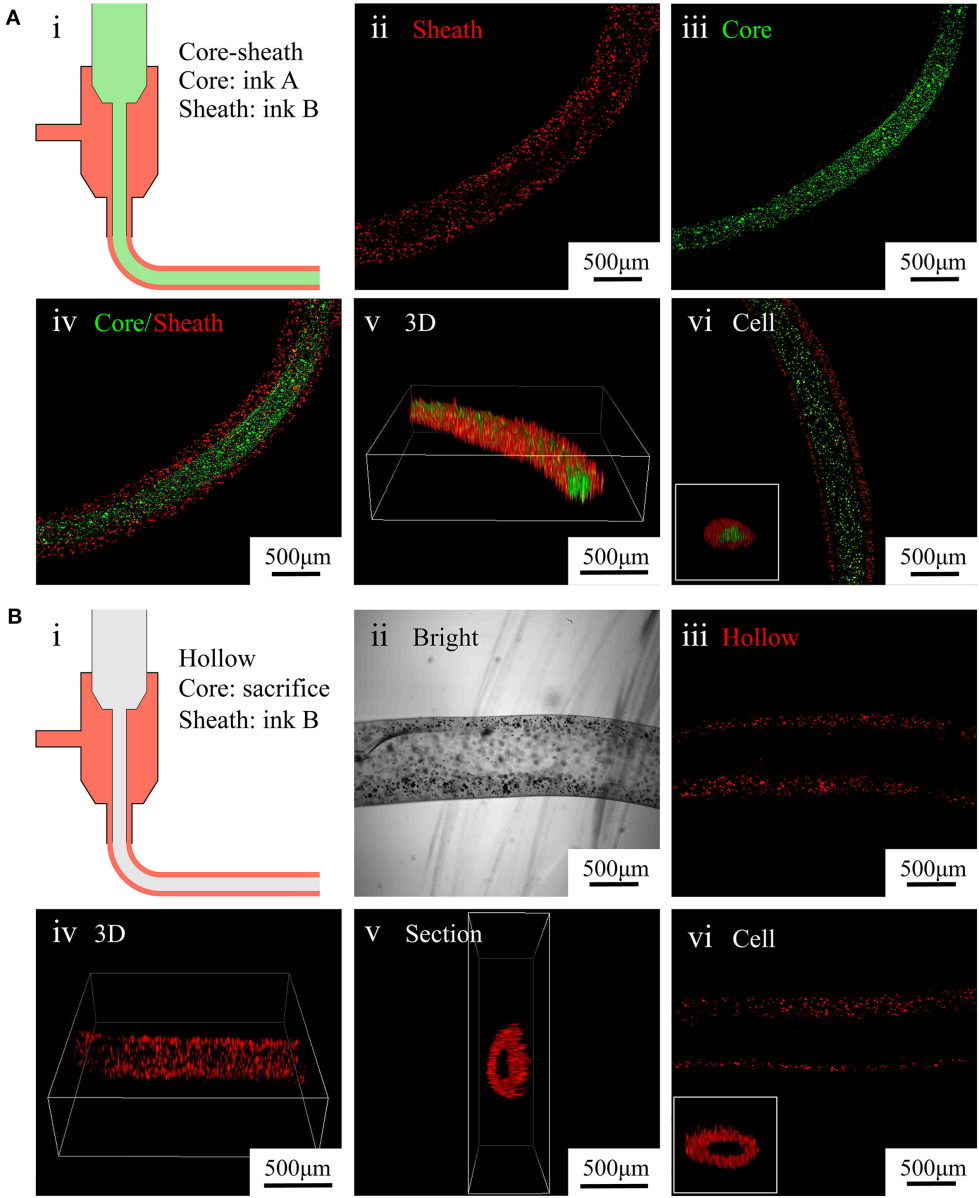
Characterization of core–sheath microfibers and hollow microfibers. **(A)** (i) Diagram of core–sheath microfiber fabrication, (ii–v) fluorescent images of core–sheath microfiber with red and green microparticles in the outer and inner layers, respectively, to show the material distribution in the microfiber, (vi) confocal images of core–sheath microfiber containing cells labeled with different dyes. **(B)** (i) Diagram of hollow microfiber fabrication, (ii–v) bright and fluorescent images of hollow fiber with red microparticles in the outer layer to show the tubular structure of the microfiber, (vi) confocal images of hollow microfiber containing cells labeled with red dye.

To fabricate core–sheath microfibers, we mixed different fluorescent microparticles or labeled cells with an alginate solution and extruded the mixture from a coaxial nozzle into the rotating solution (diagram i in Figure 4A). Then, we collected the formed core–sheath microfibers. The inner layer (green) and outer layer (red) of the microfiber presented distinct boundaries, as shown in images ii–iv in Figure 4A. The core–sheath structure with its uniform shape is shown in the 3D microfiber (image v in Figure 4A). The cell-encapsulating core–sheath microfibers (image vi in Figure 4A) shows that cells were evenly distributed in the microfiber and reflected the designed structure. By replacing the green core-ink with sacrificial gelatin (diagram i in Figure 4B), we fabricated hollow microfibers. The sheath which contained red fluorescent particles and the cavity inside the microfiber appeared in both the bright field (image ii in Figure 4B) and laser field (image iii in Figure 4B). We also observed the tubular uniform shape of the microfiber in the 3D image and section view (images iv and v in Figure 4B). Furthermore, we fabricated tubular microfibers containing red C2C12 cells, shown in image vi in Figure 4B.

### Characterization of Assembled 3D Constructs

**Figure 5:**
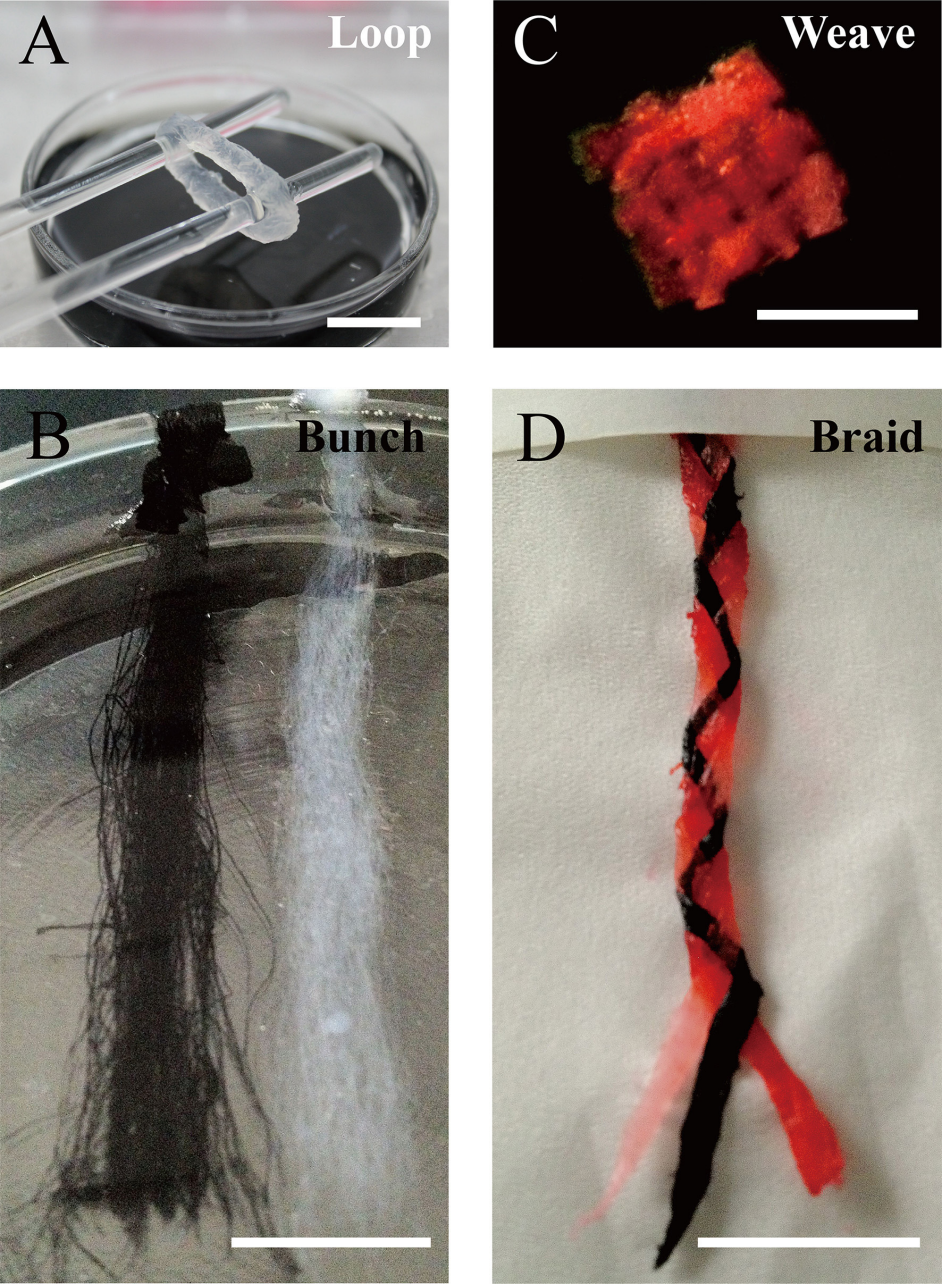
Constructs based on microfiber assembly. **(A)** Microfiber loop removed from rotor. **(B)** Fibrous modules of different colors. Microfiber constructs assembled manually by **(C)** crisscross weaving, and **(D)** spiral braiding (scale bar: 10 mm).

We designed a bunch loop to collect microfibers and build a circular engineered tissue construct. The construct was obtained by removal from the rotor at the end of the forming of hydrogel microfibers, as shown in Figure 5A. By adding black dye into the alginate solution, we obtained different fibrous modules, as shown in Figure 5B, which can be prepared for assembly. Then, we manually assembled the microfiber bunches into different constructs like crisscross weave and spiral braid, shown in Figures 5C,D, respectively.

## Discussion

For the *in vitro* construction of aligned tissue, such as muscles, nerves, and myocardia, forming both inner oriented structures and microchannels is essential for the subsequent tissue culture and functionalization. CLMs are promising units for reconstructing 3D macroscopic, spatially organized tissues. In this article, we report the rapid fabrication of various types of CLMs to construct aligned biomimetic tissue. The fabrication method is based on the fast crosslinking of alginate under the effect of divalent cations, such as calcium ions (Braccini and Pérez, 2001). As crosslinking is fast and relatively easy to operate (Gombotz and Wee, 1998), it is commonly used in the engineering of scaffolds, tissue constructs, and cell-laden constructs, like those reported in this article.

We propose the use of CLMs as modules to assemble bunch constructs, aiming to form oriented microarchitectures and cellular microenvironments that determine the subsequent development of the constructs. The hydrogel microfibers are based on alginate given its abovementioned advantages. We designed and developed the WSAC method, in which alginate-based material is wet-spun in a crosslinking bath and then gelated into the microfiber shape. The rotating flow of the crosslinking bath is created, driving the microfibers to wind up around a rotor to facilitate collection, organization, and management of the microfibers.

The microfiber diameter was regulated by factors related to crosslinking, extrusion swell, material concentration, shear rate, and flow. Regarding the formation of hydrogel microfibers, the crosslinking reaction between alginate and calcium ions is to establishes a diffusion of calcium ions driven by the ionic concentration gradient between the surface and interface. Hence, the crosslinking speed depends on the concentrations of both the alginate and calcium ions, with the speed increasing when decreasing the concentration of alginate or increasing the calcium ions (Kuo and Ma, 2001).

Moreover, given the special properties of the viscoelastic fluid, the alginate microfibers swell after extrusion (Han and Segal, 1970). The crosslinking speed and extrusion degree determine the final diameter of the microfiber. When increasing the concentration of alginate or decreasing the concentration of calcium ion, crosslinking slows down, giving more time for the microfibers to swell and consequently increasing their diameter. Furthermore, increasing the extruding speed reduces the difference between the producing speed of microfibers and the moving speed of the fluid. This promotes the adsorption and penetration of calcium ions into the hydrogel microfiber. The acceleration of the crosslinking also decreases the microfiber diameter.

When the crosslinking solution flows faster than the microfiber formation, the microfibers are dragged by the rotating flow and elongate themselves to some extent. However, when using faster agitation to enhance the rotating flow, the average microfiber diameter remains almost unaltered, indicating that the flow drag is limited. Although the magnetic rotor can provide a rotating liquid environment, the structure of the rotor is not suitable for large-scale microfiber collection. Therefore, we will devise a more stable collection equipment in future research.

Although microfibers with small diameters are generally beneficial for cells to obtain oxygen and nutrients from a culture medium, the excessive shear force during fabrication reduces the cell survival rate. The rapid forming process with controllable microfiber diameter can lead to CLMs satisfying various diameter requirements.

Usually, the breaking strength of a microfiber bundle can be assumed to follow a statistical distribution (e.g., Weibull distribution). When a bundle is loaded in parallel to the fiber direction, the weakest fiber breaks first. Then, the load redistributes among the intact fibers, resulting in an avalanche effect (Hao et al., 2018). The asynchrony of fiber breaking affects the overall strength of the bundle. Therefore, the number of fibers in a bundle decreases more sharply for bundles comprising larger fibers. The breaking asynchrony also reduces, thus increasing the tensile strength of the bundle.

Regarding cell encapsulation, alginate is unsuitable for cell culture given its lack of cell anchorage (Landers et al., 2002). To address this problem, we modified the fibrin in this study. The fibrin has been characterized by its cell signaling capabilities and facilitation of tissue regeneration (Clark, 2001). We found that fibrin in the microfibers improves the biocompatibility and is essential to promote cell viability during the *in vitro* culture. For improved cell extension and proliferation, the concentration of fibrin can be increased, but the mechanical strength of the microfiber may be compromised due to excessive fibrin, thus being unsuitable for long-term culture of constructs and subsequent tissue maturity. Besides fibrin, other biomaterials could be introduced into the current material system in upcoming studies. For example, gelatin has been demonstrated to enhance cell attachment, proliferation, and alignment (Qasim et al., 2019). Collagen, as the main component of natural extracellular matrices, has convenient biological properties and is widely used in myocardial tissue engineering (Ashammakhi et al., 2019). Moreover, GelMA, which can be crosslinked by using ultraviolet light, shows good mechanical properties and biocompatibility (Klotz et al., 2016). By adding different materials, we can improve the proposed system to provide different mechanical and biological characteristics that satisfy various requirements.

As mentioned above, the fabrication of heterogeneous microfibers or tubular microfibers is essential in tissue engineering. For example, hollow microfibers encapsulating endothelial cells can be used to construct perfusion channels inside engineered tissue and may be beneficial for vascularization. In addition, double-layer microfibers can be used to construct muscle fibers with perimysium. Especially after collection as a bunch loop, such engineered muscle fibers may be appropriate for stretch training to enhance the maturity of muscle tissue. Moreover, pre-vascularization muscle-like tissue can be fabricated by combining muscle-like CLMs and endothelial CLMs in a special order, as shown in Figure 1A. By fabricating and assembling these two types of CLMs, we can reconstruct oriented microstructures and the cell distribution of native muscle tissue. A feasible option is the braided construct consisting of three different types of microfibers, as shown in Figure 5D.

In the current WSAC system, we only used one nozzle to fabricate different microfibers. To achieve heterogeneous constructs, we may upgrade the WSAC system with multiple nozzles in future work to simultaneously fabricate and collect single-layer CLMs, hollow CLMs, and core–sheath CLMs. By changing the order or parameters of the nozzles, we can control special structures of composite constructs, which would enable the rapid construction of complex vascularized aligned tissue. By using a training bioreactor, we may obtain aligned tissue, like myocardium, with mature phenotypes. Furthermore, mature myocardium obtained from *in vitro* training can be used in the cardiotoxicity tests for drugs or the reparation of damaged hearts by surgical transplantation, which can play a great role in drug development and cardiac regeneration.

## Conclusion

We demonstrate the rapid fabrication of various CLMs that can be used to produce aligned biomimetic tissue. The proposed WSAC process to fabricate and collect hydrogel microfibers is systematically analyzed. Given the fast and mild fabrication process, cells encapsulated in alginate-fibrin microfibers are viable and healthy. We fabricated core–sheath CLMs and hollow CLMs with precise structures, possibly resembling the native tissue. Moreover, we fabricated various constructs by assembling bunch microfibers, which can be used to produce *in vitro* aligned tissue. However, the WSAC system still lacks a more stable microfiber collector and the ability to fabricate composite CLM construct without subsequent manual assembly. In the future work, we will try to upgrade the system and realize the *in vitro* fabrication and functionalization of vascularized muscle tissue.

## Data Availability Statement

The original contributions presented in the study are included in the article/supplementary material, further inquiries can be directed to the corresponding authors.

## Author Contributions

ZX and TZ conceptualized the study. BL, ML, and ZL performed the experiments. BL, ML, and YF processed the data. BL, ML, YF, ZL, TZ, and ZX wrote and reviewed the article.

## Conflict of Interest

The authors declare that the research was conducted in the absence of any commercial or financial relationships that could be construed as a potential conflict of interest.
